# Urban vs. rural differences in psychiatric diagnoses, symptom severity, and functioning in a psychiatric sample

**DOI:** 10.1371/journal.pone.0286366

**Published:** 2023-10-05

**Authors:** Lauren N. Forrest, Dan A. Waschbusch, Amanda M. Pearl, Edward O. Bixler, Lawrence I. Sinoway, Jennifer L. Kraschnewski, Duanping Liao, Erika F. H. Saunders

**Affiliations:** 1 Department of Psychiatry and Behavioral Health, Pennsylvania State University College of Medicine, Hershey, PA, United States of America; 2 Department of Medicine, Pennsylvania State University College of Medicine, Hershey, PA, United States of America; 3 Penn State Clinical and Translational Science Institute, Hershey, PA, United States of America; 4 Department of Public Health Sciences, Pennsylvania State University College of Medicine, Hershey, PA, United States of America; 5 Department of Pediatrics, Pennsylvania State University College of Medicine, Hershey, PA, United States of America; Albanian University, ALBANIA

## Abstract

**Objective:**

Identifying whether certain groups of people experience elevated rates or severities of psychiatric symptoms provides information to guide healthcare allocation. People living in urban areas have higher rates of some psychiatric disorders relative to people living in rural settings, however, it is unclear if psychiatric severity is more elevated in urban vs. rural settings. This study investigates the urban vs. rural differences in rates of psychiatric disorders and severity of psychiatric symptoms.

**Method:**

A cohort of patients (63% women, 85% White) presenting to an outpatient psychiatric treatment center in the U.S. completed patient-reported outcomes at all clinic visits as part of standard care. Rurality was determined by municipality population density. Sociodemographic characteristics, psychiatric diagnoses, trauma exposure, psychiatric symptom severity, functioning, and suicidality were compared by rural vs. urban municipality.

**Results:**

There were virtually no differences between patients living in rural vs. urban municipalities on rates of psychiatric disorders, severity of psychiatric symptoms, functional impairment, and suicidality (*p*s≥.09). The only difference was that patients living in rural municipalities had higher exposure to serious accidents than patients living in urban municipalities (*p* < .01); exposure to nine other traumatic events did not differ between groups (*p*≥.07).

**Conclusions:**

People living in urban and rural municipalities have a similar need for mental health treatment. Access to care may be one explanatory factor for the occasional rural-urban differences in rates of psychiatric disorders. In other words, if people living in rural areas can access care, their symptom presentations appear unlikely to differ from those of people living in urban areas.

## Introduction

Approximately 1 in 5 adults in the U.S. will experience a psychiatric disorder each year [1]. Psychiatric disorders cause significant burden to affected individuals, their friends and family, and the healthcare system [2]. However, the burden of mental health problems is not felt equally across the population and may not be equal between people living in urban vs. rural locations [e.g., 3–5] Some studies, including a meta-analysis, find that any psychiatric disorder, mood disorders, and anxiety disorders are more common in people living in urban settings than rural settings [4–6]. Other studies find that hazardous alcohol use, alcohol use-related harms, and PTSD are more common in rural communities as compared to urban ones [6,7], whereas some studies find that rates of all psychiatric disorders are similar between urban vs. rural dwellers [8]. Taken together, results on rural vs. urban differences in psychiatric disorder prevalence are mixed and inconclusive.

Regardless of whether psychiatric disorder prevalence differs between urban vs. rural dwellers, elevated prevalence does not necessarily mean elevated psychiatric severity. Very little research has investigated whether the psychiatric symptom severity differs between people living in urban vs. rural settings, though there are some reasons to believe that severity could differ. For example, people living in rural areas may experience increased mental health stigma, which could exacerbate symptoms and prevent seeking psychiatric treatment [9]. One study found that veterans with psychiatric disorders living in rural areas had significantly worse quality of life than veterans living in urban areas [10]. People living in rural areas may also experience greater burden from psychiatric symptoms because of limited access to psychiatric care [11,12], having to travel longer distances to see psychiatric providers [13], and systemic barriers to healthcare access (e.g., limited broadband internet access, which renders telehealth less viable [14]). Reluctance to seek treatment paired with limited and/or more difficult access to psychiatric care could result in symptoms lasting longer and/or worsening, potentially leading to elevated symptom severity in people living in rural areas.

In addition to limited research on differing psychiatric symptom severities, it is unclear if trauma exposure differs between people living in urban vs. rural settings. This is an important question because some rural primary care providers perceive that people living in rural areas have *lower* exposure to trauma than those living in urban areas [15]. If providers serving rural patients believe that traumatic experiences are infrequently experienced, providers may be less likely to assess for such experiences, resulting in inadequate mental health care and prolonged psychiatric symptoms. Contrary to the assumption that trauma exposure may be lower in patients living in rural areas, the results of the three studies investigating this question indicate that rates of traumatic experiences are similar between people living in urban vs. rural areas [8,16,17].

The current study used data from a cohort of treatment-seeking patients to address whether rates of psychiatric disorders and trauma exposure, psychiatric symptom severity, and functioning differed between people living in rural vs. urban municipalities. We hypothesized that patients residing in urban areas would have higher prevalence of mood and anxiety disorders than patients residing in rural areas [4], but that patients residing in rural areas would have greater psychiatric symptom severity than patients residing in urban areas [10].

## Method

### Participants

The Penn State Psychiatric Clinical Assessment and Rating Evaluation System (PCARES) registry is the result of the systematic collection of patient-reported outcomes from all patients presenting to an outpatient psychiatric clinic affiliated with an academic medical center in the U.S. At the time of analysis, the PCARES registry contained first-visit data from *N* = 3556 patients seen between 2/17/2015–5/30/2020. The PCARES registry also contains select, de-identified demographic information. Demographic characteristics are described in Table 1.

**Table 1:** Patient demographic characteristics stratified by urban/rural status.

	Overall		Urban		Rural		
	*N* = 3493		*n* = 3007		*n* = 486		
	*Mean*	*SD*	*Mean*	*SD*	*Mean*	*SD*	*p*
Age (year)	42.4	17.0	42.6	17.1	41.5	16.4	.18
Body mass index (kg/m^2^)	30.3	8.4	30.2	8.5	31.0	8.1	.09
**Household median income ($)**	59744	10603	60280	10778	56425	8750	**< .01**
	%	*n*	%	*n*	%	*n*	*p*
Self-reported gender							
Man	36.7	1283	36.8	1105	36.6	178	.96
Woman	63.3	2210	63.2	1902	63.4	308	
**Race**							
White	84.6	2956	83.0	2497	94.4	459	
Black or African American	5.9	206	6.7	202	0.8	4	**< .01**
Other	9.5	331	10.3	308	4.7	23	
**Ethnicity**							
Hispanic	4.4	153	4.8	143	2.1	10	
Not Hispanic Other	91.1 3.7	3210 130	91.5 3.8	2751 113	94.4 3.5	459 17	**.02**
Marital status							
Single	44.9	1568	45.4	1366	41.6	202	
Married	38.8	1354	38.0	1143	43.4	211	.08
Formerly married	16.3	571	16.6	498	15.0	73	
Primary insurance type							
Commercial	56.2	1958	56.1	1682	56.9	276	.74
Public/self-pay	43.8	1525	43.9	1316	43.1	209	
Secondary insurance type							
Commercial	13.5	464	14.5	398	13.8	66	.87
Public/self-pay	86.5	2969	86.5	2555	86.3	414	
**≥High school graduates**	90.2	4.5	90.6	4.3	87.5	5.0	**< .01**

The collection of the PCARES registry itself was considered a clinical quality improvement project that did not meet criteria for human subject research. Therefore, participant consent and Institutional Review Board (IRB) approval to collect the patient-reported outcomes were not required. However, when the de-identified PCARES data is used for research purposes, the proposed research study requires IRB approval. The current study was approved by the Pennsylvania State University IRB (Study ID #19888), and was conducted according to the ethical principles of the Declaration of Helsinki.

### Procedure

The PCARES assessment is administered at every clinic visit. Unless otherwise noted, all measures below were administered at the patient’s first visit. The assessment was modified midway through its implementation based on clinician feedback, which divides the data into “implementation phase one” and “implementation phase two.” Some measures were collected in both implementation phases, while others were collected in only one implementation phase. S1 Table shows the assessments that were administered across phases.

### Measures

#### Rural vs. urban municipality

Patients’ residential addresses were extracted from the electronic medical record (EMR) and geocoded. The geocoded addresses were merged with the Pennsylvania municipality boundaries to obtain each patient’s home Pennsylvania municipality and subsequently were cross-referenced with the rural or urban municipality designations created and maintained by the Center for Rural Pennsylvania [18]. Briefly, a Pennsylvania municipality is classified as rural when (1) the municipality population density is less than the statewide average density of 284 persons/square mile per 2010 Pennsylvania Census, or (2) the total population is less than 2,500, unless >50% of the population lives in an urbanized area as defined by the US Census Bureau. Otherwise, a municipality is classified as urban.

#### Sociodemographic characteristics

Self-reported gender, race, ethnicity, marital status, primary and secondary insurance type (commercial vs. public or self-pay), age, height, and weight were extracted from the EMR. Residential zip codes were referenced with the US Census Bureau’s 2016 American Community Survey to infer household income and education level.

#### Psychiatric diagnoses

Psychiatric diagnoses that were assigned at the first visit were extracted from the EMR.

#### Trauma exposure

The Brief Trauma Questionnaire [19] is a 10-item measure that assesses lifetime exposure to various types of traumatic experiences.

#### DSM-5 Level I cross-cutting symptom measure

The DSM-5 Level I Cross-Cutting symptom measure [20,21] is a 15-item measure of psychiatric symptoms. Participants report how much or how often, over the past two weeks, they have been bothered by each symptom on a 0 (*none* or *not at all*) to 4 (*severe* or *nearly every day*) scale. Patients were coded as “positive” on a symptom if they endorsed being mildly bothered by that symptom or experiencing a symptom several days over the last two weeks.

#### Symptom severity measures

The procedures used to assess specific symptoms were modified midway throughout the PCARES implementation. In the first phase, if a patient was coded as positive on Level 1 depression, anger, anxiety, or sleep disturbance, they completed the Level 2 symptom measures, assessed via the PROMIS Emotional Distress Depression, Anger, and Anxiety short forms and the PROMIS Sleep Disturbance short form [20,22]. In the second phase, depression was assessed with the Patient Health Questionnaire–9 (PHQ-9) [23]. Mania was assessed with the Altman Self-Rated Mania Scale [24]. Anxiety was assessed with the Generalized Anxiety Disorder–7 [25]. Suicidal thoughts and behaviors were assessed with the Columbia–Suicide Severity Rating Scale [26]. Across both phases, substance use was assessed with the Alcohol Use Disorders Identification Test, which was administered for both alcohol and other substances [27].

#### Functioning

Both implementation phases included the World Health Organization Disability Assessment Scale-2.0 [28]. This is a 36-item measure assessing impaired functioning across several domains due to physical or mental health concerns in the last 30 days.

### Data analytic plan

The current report included all available data for each measure (i.e., pairwise deletion). All analyses were performed in SAS version 9.4. Chi-square, t-tests, and Cochran-Mantel-Haenszel tests compared demographic and clinical characteristics by rural vs. urban municipality. For categorical outcomes, effect sizes were indicated by the difference in proportions (% rural–% urban). For continuous outcomes, effect sizes were indicated by Cohen’s *d* (rural *mean–*urban *mean* / overall *SD*).

## Results

Descriptive statistics by rural vs. urban municipality are presented in Table 1. Rural and urban patients had similar *mean* age and body mass index, and similar proportions of people based on self-reported gender, marital status, primary insurance type, and secondary insurance type. However, compared to urban municipalities, rural municipalities had lower median household income (*p* < .01), higher proportion of White-identified people (*p* < .01), higher proportion of non-Hispanic-identified people (*p* = .02), and lower education (*p* < .01).

Clinical characteristics by urban vs. rural municipality are shown in Tables 2–4. Patients living in rural vs. urban municipalities had similar proportions of all psychiatric disorders (*p*s≥ .12; Table 2). A significant difference was found for only one of 10 traumatic events, such that people living in rural municipalities had higher exposure to serious accidents than patients living in urban municipalities (*p* < .01; Table 2). Rural and urban municipalities had similar proportions of patients screening positive on all DSM Level 1 cross-cutting symptom measures (*p*s≥.10; Table 3). Patients living in rural vs. urban municipalities had similar severities of depression (*p*s = .37-.72), anxiety (*p*s = .60-.74), sleep problems (*p* = .21), anger (*p* = .51), mania (*p* = .22), alcohol use (*p* = .38), substance use (*p* = .65), and suicidal thoughts and behaviors (*p*s≥.12; Table 4). Patients living in rural vs. urban municipalities had similar scores on all indices of functioning (*p*s≥.33; Table 4).

**Table 2:** Psychiatric diagnoses by urban/rural status.

	Overall *N* = 3493		Urban *n* = 3007		Rural *n* = 486			
	%	*n*	%	*n*	%	*n*	Effect size*	*p*
Major depressive disorder–Lifetime	41.1	1432	40.7	1221	43.5	211	2.8%	.24
Major depressive disorder–Current	37.7	1313	37.2	1116	40.6	197	3.4%	.15
Any Bipolar-Lifetime	10.2	356	10.4	311	9.3	45	-1.1%	.46
Bipolar I disorder–Lifetime	3.0	104	3.2	95	1.9	9	-1.3%	.12
Bipolar II disorder–Lifetime	3.0	106	3.0	91	3.1	15	0.1%	.94
Bipolar disorder NOS–Lifetime	4.4	154	4.4	132	4.5	22	0.1%	.89
Generalized anxiety disorder	21.6	752	21.2	637	23.7	115	2.5%	.22
Panic disorder	5.3	183	5.4	163	4.2	20	-1.2%	.23
Social phobia	2.4	83	2.4	72	2.3	11	-0.1%	.86
Psychotic disorder–Lifetime	3.2	112	3.4	102	2.1	10	-1.3%	.12
Obsessive compulsive disorder	3.1	108	3.2	95	2.7	13	-0.5%	.57
Post-traumatic stress disorder	6.3	219	6.3	189	6.2	30	-0.1%	.92
Antisocial personality disorder	0.1	2	0.1	2	0.0	0	-0.1%	N/A
Eating disorder	1.0	35	1.1	32	0.6	3	-0.5%	.47
Alcohol use disorders	1.6	54	1.7	50	0.8	4	-0.9%	.16
Substance use disorders	1.6	56	1.6	47	1.9	9	0.3%	.64
Opioid use disorder	0.3	10	0.2	7	0.6	3	0.4%	.15
Somatic disorder	0.7	24	0.6	18	1.2	6	0.6%	.13
Other personality disorder	1.2	43	1.2	37	1.2	6	0.0%	.99
Autism spectrum disorder	5.6	196	5.4	161	7.2	35	1.8%	.10
Attention deficit–hyperactivity disorder	7.0	243	7.1	212	6.4	31	-0.7%	.58
	*Mean*	*SD*	*Mean*	*SD*	*Mean*	*SD*	Effect size*	*p*
Number of diagnoses^†^	1.5	1.1	1.5	1.1	1.5	1.2	0.04	.33
	Overall *n* = 1102		Urban *n* = 971		Rural *n* = 131			
Brief Trauma Questionnaire	%	*n*	%	*n*	%	*n*	Effect size*	*p*
War zone, casualty	2.6	28	2.5	24	3.1	4	0.6%	.77
Serious accident	**26.9**	**290**	**25.5**	**243**	**37.9**	**47**	12.4%	**< .01**
Major disaster	15.1	164	15.1	146	14.4	18	-0.7%	.83
Life-threating illness	16.2	175	15.8	151	18.9	24	3.1%	.38
Child abuse	26.9	292	26.8	256	27.7	36	0.9%	.83
Physical attack	27.8	300	28.7	273	21.1	27	-7.6%	.07
Unwanted sex	34.3	371	34.1	325	35.7	46	1.6%	.73
Seriously injured	21.7	234	21.7	206	21.9	28	0.2%	.96
Others died violently	20.3	221	20.0	192	22.5	29	2.5%	.51
Others injured seriously	24.3	258	24.3	227	24.4	31	0.1%	.97
Patients with > = 1 “yes”	74.9	825	74.2	720	80.2	105	6.0%	.14
	*Mean*	*SD*	*Mean*	*SD*	*Mean*	*SD*	Effect size^†^	*p*
Number of “Yes”	2.1	2.0	2.1	2.0	2.2	1.9	0.05	.55

*Note*. NOS = Not otherwise specified.

*Effect size for categorical variable = rural %–urban %, effect size for continuous variable = (rural *mean–*urban *mean*)/overall *SD*.

† # of diagnoses was calculated as the sum of all clinical diagnoses, except “Any Bipolar-Lifetime”.

**Table 3:** Percentage of the sample with DSM-5 Level 1 Cross-Cutting Symptom Measure screener that was positive stratified by urban/rural status.

	Overall *n* = 2307		Urban *n* = 1997		Rural *n* = 310			
DSM Level I^†^	%	*n*	%	*n*	%	*n*	Effect size*	*p*
Depression	81.5	1876	81.3	1621	82.5	255	1.2%	.60
Anger	69.4	1595	68.9	1371	72.7	224	3.8%	.17
Mania	42.0	954	41.9	826	42.2	128	0.3%	.91
Anxiety	83.0	1905	83.0	1649	82.9	256	-0.1%	.94
Somatic	69.8	1593	69.8	1381	69.3	212	-0.5%	.85
Suicide	29.5	676	25.9	573	33.6	103	4.6%	.10
Psychosis	14.8	340	14.9	295	14.7	45	-0.2%	.94
Sleep disturbance	67.4	1547	67.2	1335	68.8	218	1.6%	.57
Memory	44.8	1026	44.9	891	44.3	135	-0.6%	.84
OCD	45.8	1040	45.7	897	46.6	143	0.9%	.76
Dissociation	35.2	802	35.5	701	33.2	101	-2.3%	.43
Personality	64.4	1468	64.4	1271	64.4	197	0.0%	.99
Alcohol use	4.9	112	5.1	101	3.6	11	-1.5%	.26
Tobacco use	22.0	502	21.6	428	24.3	74	2.7%	.30
Substance use	6.6	151	6.8	134	5.6	17	-1.2%	.44
	*Mean*	*SD*	*Mean*	*SD*	*Mean*	*SD*	Effect size*	*p*
Total score	30.4	16.4	30.4	16.4	30.4	16.2	0.00	.97
Symptom domains, #	6.5	3.2	6.5	3.2	6.6	3.2	0.03	.82

^†^ Scoring algorithm in Supplemental Table 2.

*: Effect size for categorical variable = % (rural)—% (urban).

Effect size for continuous variable = [Mean (rural)–Mean (urban)]/SD (overall).

**Table 4:** Psychiatric symptom severity and functioning stratified by urban/rural status.

	Overall			Urban			Rural				
	*n*	*Mean*	*SD*	*n*	*Mean*	*SD*	*n*	*Mean*	*SD*	Effect size*	*p*
Phase 1 symptom severity measures											
Depression T-score	1232	65.1	8.7	1054	65.0	8.6	178	65.3	9.6	0.03	.72
Anxiety T-score	1232	65.9	8.8	1054	65.8	8.8	178	66.1	8.8	0.03	.74
Sleep T-score	1232	62.8	8.0	1054	62.7	8.0	178	63.7	7.7	0.13	.21
Anger T-score	1232	62.9	10.5	1054	62.8	10.6	178	63.4	10.5	0.06	.51
Phase 2 symptom severity measures											
Depression (PHQ-9)	2756	10.7	7.1	2387	10.8	7.1	369	10.4	7.1	-0.06	.37
Anxiety (GAD-7)	2585	9.5	6.4	2238	9.4	6.4	347	9.6	6.4	0.03	.60
Mania (ASRM)	2515	4.47	4.24	2160	4.51	4.22	355	4.21	4.32	-0.07	.22
	*n*		*%*	*n*		*%*	*n*		*%*	Effect size*	*p*
Suicide (C-SSRS^†^)												
None	1801		69.7	1545		69.2	256		73.1	3.9%		
Wished dead	264		10.2	233		10.4	31		8.9	-1.5%		
Think of killing self	87		3.4	74		3.3	13		3.7	-0.4%		
How may kill self	97		3.8	87		3.9	10		2.9	-1.0%		.12
Intention of act	29		1.1	23		1.0	6		1.7	0.7%		
Work out details	19		0.7	15		0.7	4		1.1	0.4%		
Attempts/harm self	175		6.8	153		6.9	22		6.3	-0.6%		
“Yes” past 3 mo.	13		7.4	13		8.5	0		0.0	-8.5%		N/A
Start to kill yourself	112		4.3	104		4.7	8		2.3	-2.4%		
“Yes” past 3 mo.	31		27.5	31		29.5	0		0.0	-29.5%		N/A
	*n*	*Mean*	*SD*	*n*	*Mean*	*SD*	*n*	*Mean*	*SD*	Effect size*	*p*
Continuous score	2584	1.1	2.1	2234	1.1	2.1	350	0.9	1.9	-0.10	.09
Phase 1 and Phase 2 measures											
Alcohol/substance use (AUDIT)											
Alcohol use	1104	3.0	4.2	973	3.1	4.2	131	2.7	4.0	-0.10	.38
Substance use	1104	1.5	2.6	973	1.5	2.6	131	1.6	2.9	0.04	.65
WHODAS											
Cognition	3322	29.6	24.6	2856	29.6	24.6	466	29.6	24.1	0.00	0.99
Mobility	3322	28.3	31.0	2856	28.0	30.9	466	29.4	31.9	0.05	0.39
Self-care	3322	14.2	22.5	2856	14.3	22.7	466	13.3	21.2	-0.04	0.39
Getting along	3322	34.6	29.9	2856	34.7	29.8	466	34.1	30.5	-0.02	0.65
Life activities	3322	47.3	28.2	2856	47.3	28.0	466	47.3	29.0	0.00	0.99
Participation	3322	46.0	28.3	2856	45.7	28.2	466	47.1	28.9	0.05	0.33
Total score	3322	31.8	21.2	2856	31.8	21.1	466	32.1	21.5	0.01	0.80

ASRM = Altman Mania Rating Scale, PHQ-9 = Patient Health Questionnaire– 9, GAD-7 = Generalized Anxiety Disorder -7, AUDIT = Alcohol Use Disorders Identification Test, C-SSRS = Columbia Suicide Severity Rating Scale, WHODAS = World Health Organization Disability Assessment Scale 2.0.

† C-SSRS score equals to the question number of the “highest” (i.e. most severe suicidal thoughts or behaviors) question with “Yes” as the answer. The column percentages/Ns are mutually exclusive. The prevalence of “Yes within 3 months” for Q6 and Q7 were calculated WITHIN those who scored 6 or 7, respectively. For example, among the 175 patients who scored “6” (“Yes” on attempted to kill or harm yourself), 7.4% (n = 13) of them took actions within the last 3 months.

* Effect size for categorical variable = rural %–urban %, effect size for continuous variable = (rural *mean*–urban *mean*)/overall *SD*.

## Discussion

There were virtually no differences between urban vs. rural dwellers on rates of psychiatric diagnoses, symptom severities, suicidal thoughts and behaviors, or functional impairment. The only significant difference was that people living in rural municipalities had higher exposure to one of ten traumatic experiences than people living in urban municipalities. The lack of significant differences on diagnoses and symptom severities could indicate that access to care may be one explanatory factor for the occasional differences in rates of psychiatric disorders observed in population-based studies. In other words, if people living in rural areas can access care, their symptom presentations may be unlikely to differ from those of people living in urban or suburban locations.

The lack of systematic differences on psychiatric symptoms between patients living in urban vs. rural municipalities indicates that rural and urban dwellers have a similar need for psychiatric treatment. This can be viewed as both a glass half full and a glass half empty. On one hand, rurality in and of itself does not appear to indicate a need for differential psychiatric assessments or treatments [e.g., 15]. On the contrary, if patients living in rural areas can access mental health care, the current results suggest that providers can treat these patients using current best practices, such as assessing the intersecting aspects of a patient’s environment, history, and social systems that contribute to mental health [29,30]. Indeed, although people living in urban vs. rural locations may have different values and identities that can impact mental health [e.g., 31,32], rural and urban dwellers also share substantial commonalities regarding mental health (aside from access to care). Although “diseases of despair,” which include depression, drug overdose, alcohol-related liver disease, and suicidal thoughts and behaviors, have often been associated with rural America [33], population-based data indicate that diseases of despair impact people regardless of their place of residence on the rurality continuum [34]. Further, a qualitative study found that community members living in rural and urban locations had shared beliefs in their perception of causal factors driving despair in their communities [35].

On the other hand, despite a similar need for psychiatric treatment in rural vs. urban residents, limited access to mental health care in rural areas is well documented [29]. Rural health advocates have long sought to increase rural access to mental health care, such as through the creation of the Behavioral Health Workforce Education and Training program [36] and deployment of telehealth services [37,38]. Unfortunately, disparities in access to mental health care still persist [29,38]. Dismantling these barriers to mental health care likely requires a complex and multi-modal process of examining how geographic location intersects with socioeconomic status, race, ethnicity, sexual orientation, educational opportunities, occupational conditions, and community changes, and how these factors are situated within systems, institutions, communities, and public policies [29,30,39]. The current results do not provide suggestions for how to begin solving this problem. Rather, they provide further data supporting that people living in rural areas have a similar need for psychiatric treatment as their urban counterparts and underscore the need to increase rural access to mental health treatment.

The study strengths are as follows. We administered transdiagnostic symptom measures to a large sample of patients. This is important because much of the existing rural mental health disparities research has assessed diagnoses only, whereas transdiagnostic assessment is in line with contemporary developments in psychopathology research and classification (e.g., Research Domain Criteria) [40]. The findings reflect real-world data from a cohort of patients seeking psychiatric treatment from the same academic medical center, thereby increasing generalizability.

Several limitations are of note. First, although the study’s real-world data may enhance generalizability, some aspects of these data are not ideal for research. For example, data are missing for several reasons, including having two phases of assessment implementation and because of aspects of the measurement-based care implementation (e.g., screening negatively on the DSM Level 1 Cross-Cutting Symptom Measure means that a Level 2 Measure is not completed). Second, a different pattern of results could emerge with a community- or population-based sample where participants are stratified both on rurality and whether they have received psychiatric treatment [41,42]. Third, psychiatric diagnoses were assigned based on provider interview/judgment, and not assigned based on a standardized interview. Fourth, we defined rurality based on patients’ municipalities. Rurality can also be coded by county. In the current report, coding rurality by county yielded a highly similar pattern of results (data not shown). Regardless of coding rural status by municipality or county, both definitions are dichotomous, and yet rurality exists on a continuum [e.g., 43]. Larger-scale data that quantifies the rurality continuum is needed.

In sum, we found very little evidence for urban vs. rural differences in rates of psychiatric disorders, trauma exposure, psychiatric symptom severity, severity of suicidal thoughts and behaviors, and functional impairment. Results underscore that people living in urban and rural areas have an equal need for mental health treatment.

## Supporting information

S1 Table PCARES battery at implementation Phase 1 and implementation Phase 2. (DOCX)

S2 Table DSM Level I symptom algorithms. (DOCX)
