# Supplementary material for: Urban vs. rural differences in psychiatric diagnoses, symptom severity, and functioning in a psychiatric sample
Source: PLoS One. 2023 Oct 5;18(10):e0286366. doi: 10.1371/journal.pone.0286366 (PMC10553337; doi:10.1371/journal.pone.0286366)
Supplement: S1 Table — (DOCX) [file pone.0286366.s001.docx]

**S1 Table.** PCARES Battery at Implementation Phase 1 and Implementation Phase 2

|  | Phase 1 | Phase 2 |
| --- | --- | --- |
| DSM-5 Level 1 Cross-Cutting Symptom Measure | x | x |
| Alcohol Use Disorders Identification Test (AUDIT) | x | x |
| World Health Organization Disability Assessment Scale 2.0 | x | x |
| Mini-International Neuropsychiatric Interview 6.0 | x |  |
| PROMIS Emotional Distress – Depression | x |  |
| PROMIS Emotional Distress – Anger | x |  |
| PROMIS Emotional Distress – Anxiety | x |  |
| PROMIS Sleep Disturbance – Short Form | x |  |
| Brief Trauma Questionnaire |  | x |
| Patient Health Questionnaire (PHQ-9) |  | x |
| Generalized Anxiety Disorder Scale (GAD-7) |  | x |
| Altman Self-Rating Mania Scale (ASRM) |  | x |
| Columbia-Suicide Severity Rating Scale |  | x |
