# Supplementary material for: Urban vs. rural differences in psychiatric diagnoses, symptom severity, and functioning in a psychiatric sample
Source: PLoS One. 2023 Oct 5;18(10):e0286366. doi: 10.1371/journal.pone.0286366 (PMC10553337; doi:10.1371/journal.pone.0286366)
Supplement: S2 Table — (DOCX) [file pone.0286366.s002.docx]

**S2 Table.** DSM Level I Symptom Algorithms

| Symptom Domain | Question numbers | Cutoff |
| --- | --- | --- |
| Depression | Q1, Q2 | ≥ 2 |
| Anger | Q3 | ≥ 2 |
| Mania | Q4, Q5 | ≥ 2 |
| Anxiety | Q6, Q7, Q8 | ≥ 2 |
| Somatic | Q9, Q10 | ≥ 1 |
| Suicidal | Q11 | ≥ 1 |
| Psychotic | Q12, Q13 | ≥ 1 |
| Sleep disturbance | Q14 | ≥ 2 |
| Memory | Q15 | ≥ 2 |
| OCD | Q16, Q17 | ≥ 2 |
| Dissociation | Q18 | ≥ 2 |
| Personality | Q19, Q20 | ≥ 2 |
| Alcohol use | Q21 | ≥ 2 |
| Tobacco use | Q22 | ≥ 2 |
| Substance use | Q23 | ≥ 2 |

A patient is considered as having the symptom if **any** of the question within the domain is above the cutoff. For example, a patient is considered as having level I depression, if either Q1 or Q2 was scored ≥ 2.
